# A Multi-country Study of the Household Willingness-to-Pay for Dengue Vaccines: Household Surveys in Vietnam, Thailand, and Colombia

**DOI:** 10.1371/journal.pntd.0003810

**Published:** 2015-06-01

**Authors:** Jung-Seok Lee, Vittal Mogasale, Jacqueline K. Lim, Mabel Carabali, Chukiat Sirivichayakul, Dang Duc Anh, Kang-Sung Lee, Vu Dinh Thiem, Kriengsak Limkittikul, Le Huu Tho, Ivan D. Velez, Jorge E. Osorio, Pornthep Chanthavanich, Luiz J. da Silva, Brian A. Maskery

**Affiliations:** 1 International Vaccine Institute, Seoul, South Korea; 2 Department of Tropical Pediatrics, Faculty of Tropical Medicine, Mahidol University, Bangkok, Thailand; 3 National Institute of Hygiene and Epidemiology, Hanoi, Vietnam; 4 Khanh Hoa Health Services, Nha Trang, Vietnam; 5 Programa de Estudio y Control de Enfermedades Tropicales (PECET), Universidad de Antioquia, Medellín, Colombia; 6 Department of Pathobiological Sciences, University of Wisconsin, Madison, Madison, Wisconsin, United States of America; Brandeis University, UNITED STATES

## Abstract

**Background:**

The rise in dengue fever cases and the absence of dengue vaccines will likely cause governments to consider various types of effective means for controlling the disease. Given strong public interests in potential dengue vaccines, it is essential to understand the private economic benefits of dengue vaccines for accelerated introduction of vaccines into the public sector program and private markets of high-risk countries.

**Methodology/Principal Findings:**

A contingent valuation study for a hypothetical dengue vaccine was administered to 400 households in a multi-country setting: Vietnam, Thailand, and Colombia. All respondents received a description of the hypothetical dengue vaccine scenarios of 70% or 95% effectiveness for 10 or 30 years with a three dose series. Five price points were determined after pilot tests in order to reflect different local situations such as household income levels and general perceptions towards dengue fever. We adopted either Poisson or negative binomial regression models to calculate average willingness-to-pay (WTP), as well as median WTP. We found that there is a significant demand for dengue vaccines. The parametric median WTP is $26.4 ($8.8 per dose) in Vietnam, $70.3 ($23.4 per dose) in Thailand, and $23 ($7.7 per dose) in Colombia. Our study also suggests that respondents place more value on vaccinating young children than school age children and adults.

**Conclusions/Significance:**

Knowing that dengue vaccines are not yet available, our study provides critical information to both public and private sectors. The study results can be used to ensure broad coverage with an affordable price and incorporated into cost benefit analyses, which can inform prioritization of alternative health interventions at the national level.

## Author Summary

Dengue is complicated. There are four serotypes of the dengue virus, and dengue infection occurs in almost all age groups. Infection with one serotype provides life-long immunity to that specific serotype but does not protect against the other three serotypes. Unlike other diseases which already have preventable vaccines developed, currently there are no commercially available vaccines for dengue fever. Even if the first vaccine becomes available, it is expected that there will be a limited number of vaccine doses available in the first few years. Due to the increase in dengue fever cases, there is already huge public and private interest in potential dengue vaccines. This study reports the household willingness-to-pay for a hypothetical dengue vaccine in three dengue endemic countries. We found that household demand is strongly related to price and income. It was also observed that more than half of the study populations are willing to pay for vaccines when price is lower than the median estimates reported here. This study may contribute to a more effective decision on dengue vaccine introduction.

## Introduction

Dengue fever is a major public health concern in South-East Asia and South America. Dengue virus is transmitted to humans by Aedes mosquitoes. Clinical presentation ranges from self-limited, mild febrile illness to classic dengue fever (DF) to the more severe form of illness, dengue hemorrhagic fever (DHF). The global burden of dengue has increased dramatically in the past five years, and presently, DF and DHF are recognized as a major cause of mortality and morbidity in tropical and sub-tropical countries[1,2]. The recent study shows that there are 96 million apparent and 294 million inapparent dengue infections occurring yearly, and the total 390 million infections are more than three times the previous estimate of the World Health Organization (WHO)[3–6]. At present, there is no specific treatment for dengue infection. Mosquito control prevention efforts have not been sufficient to control the disease. Vaccine development is still in progress.

The Dengue Vaccine Initiative (DVI) has conducted extensive multidisciplinary dengue fever studies for decision makers in three countries: Vietnam, Thailand, and Colombia. In Vietnam, annual disease incidence is reported to be 145/100,000 population according to the national surveillance system in 2010[7]. Because extensive studies in Vietnam are lacking, a better understanding of dengue and its impact is necessary for disease control, especially in making decisions in regard to future implementation of dengue vaccines. In Thailand, DF/DHF has steadily increased in both incidence and range of distribution despite mosquito control efforts. The dengue incidence rate in Thailand was estimated to be 177/100,000 population in 2010[8]. Dengue epidemiology in Thailand can be characterized by the circulation of all four dengue serotypes and the presence of a well-established national dengue surveillance system. Colombia has seen a significant increase in cases of DF/DHF during the last 10 years, with epidemic waves occurring every 3–4 years. In 2010, Colombia experienced the largest recorded epidemic with the dengue incidence rate of 685/100,000 population[9]. All four dengue serotypes are found in Colombia, and in significant contrast to Asian countries, the disease occurs in people of all ages.

The rising tide of the Dengue Fever and its associated morbidities underscore the need for vaccines against dengue[10–12], but there continues to be a lack of economic assessment on dengue fever vaccines. Vaccines currently under development may significantly reduce the burden of disease. The three countries mentioned above are each candidates to become early adopters of future dengue vaccines. However, like many other low- and middle-income countries, these countries will face decisions on whether and how to incorporate new and potentially expensive vaccines within their budget-constrained national vaccination programs. Therefore, understanding the private economic benefits of potential dengue vaccines is necessary for accelerated introduction of vaccines into the public sector program and private markets of high-risk countries.

## Methods

### Research design

**Fig 1:**
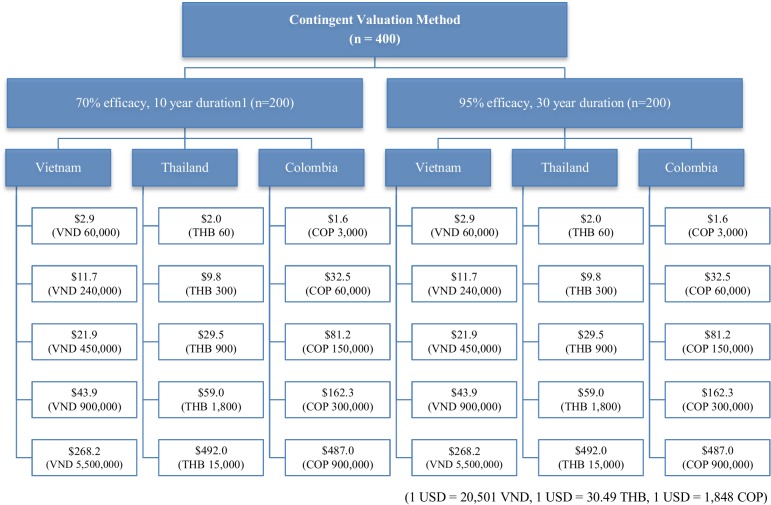
Study design.

To estimate household demand and WTP for hypothetical vaccines against dengue infection, we administered a study questionnaire to 400 households in each of three countries (Fig 1). All respondents (*N* = 400) at each site received a description of the hypothetical dengue vaccine scenarios of 70% or 95% efficacy for 10 or 30 years. Five pre-assigned prices were determined after performing pilot tests (40 pretest interviews) and open-ended focus group discussions. In this analysis, the dichotomous choice method was adopted. Unlike other CV studies such as open-ended, bidding game, and payment card which have shown incentive compatibility problems, dichotomous choice eases the burden on the respondents, decreasing the number of protest answers[13]. Respondents were asked if they would be willing to buy a vaccine for their youngest child and other household members at randomly pre-assigned prices. Interviewers reminded respondents of their budget constraints and mentioned that there were no right or wrong answers.

We adopted a *time-to-think* approach in Colombia and Vietnam, which gives respondents time to deliberate their decision on whether they would like to purchase a new vaccine. In previous split sample studies[14–17], respondents tended to demand significantly fewer vaccines when provided with more time to think about their purchasing decision compared to respondents that completed interviews in one sitting. In Colombia and Vietnam, respondents received general information on dengue illness, risk factors and a hypothetical vaccine. They were instructed to consider their vaccine purchasing decision overnight. The *time-to-think* design was not implemented in Thailand because of cost and logistical issues. Instead, respondents completed the entire survey in one interview.

### Site selection and sampling procedure

The three sites were selected in support of multidisciplinary research goals of the Dengue Vaccine Initiative. In addition to economic studies, these sites were chosen to provide an in-depth picture of dengue epidemiology and transmission within high risk populations of the chosen countries. All three sites share several common characteristics—high levels of dengue virus transmission; stable population with low rates of migration and high rates of ethnic homogeneity; sites are easily accessible with good health services; and local dengue control officers and provincial public health officials are exceptionally motivated and committed to dengue research. Interviews were administered to the head /senior of selected households.

#### Vietnam (Mar—Aug, 2012)

Khanh Hoa province is located on the South central coast of Vietnam. Our catchment area is Nha Trang city which is the capital of Khanh Hoa province. Dengue fever is known to be highly endemic in the southern part of Vietnam, with a reported dengue incidence for Nha Trang city of 358/100,000 in 2010[18]. There are 27 communes in Nha Trang. In collaboration with Nagasaki University, census information collected in 16 communes was shared and used as baseline demographic data in the DVI’s study in Nha Trang. The study was conducted in three rural and three urban communes which were randomly selected among 16 communes.

#### Thailand (Sep—Nov, 2012)

Ratchaburi province ranks among the top ten provinces of Thailand for dengue incidence rates, and among Ratchaburi’s 10 districts, dengue transmission in Bang Phae district is consistently higher than most others. The reported annual incidence of dengue ranged from 55.8–638.3/100,000 for the district between 2000 and 2010[19]. Among 65 villages in Bang Phae district, a village with less than 1,000 residents was considered as one cluster. If village population was greater than 1,000, the village was subdivided into two clusters. The clusters were stratified based on rural and urban areas, and 400 households were approached from 40 randomly selected clusters.

#### Colombia (Mar—Oct, 2012)

The metropolitan area of Medellin in the state of Antioquia is the second largest city in Colombia. As in the rest of the country, dengue virus is endemic in Medellin, with outbreaks occurring periodically and increasing in frequency significantly over the past 20 years. The reported annual incidence of dengue varied from 161-745/100,000 between 2003 and 2010[20]. The recent high dengue-transmission year was 2010. Santa Cruz is one of 16 districts within the city of Medellin and has 11 neighborhoods. Systematic sampling was conducted such that every seventh household was selected, and 400 households were taken from all of the neighborhoods.

### Survey questionnaire

The questionnaire consists of six sections. The first section collected demographic information about the respondent and members of the household. The second section asked about respondent’s perception and experience regarding dengue fever, including activities undertaken by the household to reduce their risk of dengue infection. The third section included information for the respondents on general conditions of dengue fever including how the disease is transmitted and dengue fever risk may be mitigated through community-wide efforts. This section also recorded previous vaccination history, and provided a description of the hypothetical dengue vaccine, including efficacy and duration of protection which can be found in S2 Text and S2 Fig. A series of questions were asked to ensure that respondents had understood how the vaccine works. In the fourth section, household demand was collected. For example, the first WTP question was framed as: “Suppose that the total cost for the dengue fever vaccine would be VND 450,000 for three dose needed for one person. Would you buy this vaccine for your youngest child?” To access household WTP, the additional question was followed: “Suppose that this dengue fever vaccine costs VND 450,000 for the 3-dose series required for each person (same price for adults and children), how many people in your household (not including your youngest child) would you be willing to purchase vaccines for?…Who would you buy this vaccine for?” The responses were recorded in a table which is linked to the demographic information in the first section. Respondents who said that they would not buy the vaccine at the specified price were asked if they would take the vaccine if it were provided free. For those who did not want to take a free vaccine or pay any positive price, an additional question was posed to see why they would not take the vaccine. The respondents refused to take the vaccine because they did not think that vaccines are safe or prevent the disease were determined as out-of-market respondents and did not proceed to the next step. In the fifth section, socioeconomic information was collected, such as education, occupation, income, and economic status. The sixth section included questions regarding the *time-to-think* approach. Pilot studies, focus group discussion and pre-final questionnaires, were conducted in each of the three studies to refine the survey instrument and to help determine an appropriate set of prices for each setting. Results from the pilot studies were not included in this analysis.

### Modeling

Our underlying economic model assumes that respondents maximize their household utility, subject to their budget constraints. Vaccines are one of many purchases that can be used to build health capital and household health is one of many competing spending choices. Household vaccine demand is a non-negative integer value and the number of vaccines demanded (dependent variable) can be estimated as a function of vaccine price, efficacy, household perceptions of dengue severity and likelihood, as well as household socio-economic characteristics. Count models are suitable for our household demand analysis because the count model estimates non-negative integer values and specifies the quantity demanded with a mean that is dependent on exogenous variables[13,21]. The Poisson or its variants (e.g., negative binomial) typically takes the exponential form for expected demand, and the Poisson probability density function can be written as

$$\mathrm{Pr}\left({\text{x}}_{\text{i}}=\text{n}\right)=\frac{{\text{e}}^{-\lambda_{\text{i}}}\lambda_{\text{i}}^{\text{n}}}{\text{n}!},\text{n}=0,1,2\ldots$$

where *n* is observed demand, and λ_i_ is the mean, λ_i_ = exp(z_i_β). For the Poisson model, the mean is equal to the variance of the distribution. If the variance is greater than the mean, the model is mis-specified due to overdispersion[22,23] (S1 Text). Overdispersion may not affect the coefficient estimates significantly, but causes standard errors to be underestimated. For this reason, the Z-score test and the boundary likelihood ratio test were performed to test for overdispersion for each country[22]. The negative binomial technique relaxes the assumption of equality of the mean and variance by adding a gamma distributed error term[24]. A common version of the negative binomial model is as follows:

$$\begin{array}{c} \text{E}\left({\text{x}}_{\text{i}}{\mathrm{|z}}_{\text{i}}\beta \right)=\lambda_{\text{i}}=\exp\left({\text{z}}_{\text{i}}\beta \right)\\ \log\left(\text{E}\left({\text{x}}_{\text{i}}\right)\right)={\text{z}}_{\text{i}}\beta +\theta_{\text{i}} \end{array}$$

where θ_i_ represents unobserved individual differences (or unobserved heterogeneity).

$$\mathrm{Pr}\left({\text{x}}_{\text{i}}\right)=\frac{\Gamma \left({\text{x}}_{\text{i}}+\frac{1}{\alpha }\right)}{\Gamma \left({\text{x}}_{\text{i}}+\frac{1}{\alpha }\right)\Gamma \left(\frac{1}{\alpha }\right)}{\left(\frac{\frac{1}{\alpha }}{\frac{1}{\alpha }+\lambda_{\text{i}}}\right)}^{\frac{1}{\alpha }}{\left(\frac{\lambda_{\text{i}}}{\frac{1}{\alpha }+\lambda_{\text{i}}}\right)}^{{\text{x}}_{\text{i}}}$$

where λ_i_ = exp(z_i_β). The mean of the negative binomial distribution is E(x_i_) = λ_i_ = exp(z_i_β). However, now the variance of the dependent variable is V(x_i_) = λ_i_(1 + αλ_i_). The parameter α can be interpreted as the overdispersion parameter. When α is equal to zero, the variation becomes equal to the mean, and the distribution can be modelled by Poisson regression. However, if α is greater than zero, overdispersion exists, and the Poisson model is rejected in favor of the negative binomial model[13] (S1 Fig). Standard errors were corrected for the cluster sampling procedure to improve the accuracy of the estimates.

Model validation is critical to check whether a model is appropriate and useful. There are several statistics which estimate how well the model fit the data, how much error was in the model[25]. Mean Absolute Deviation (MAD) and Mean Squared Prediction Error (MSPE) were used to estimate how well the model fit the data [24–26]. MAD provides a measure of the average mis-prediction of the model, and MSPE is typically used to assess the error associated with a validation or external dataset[26].

$$\begin{array}{c} \text{MAD}=\frac{\sum_{i=1}^{n}\left|{\hat{Y}}_{i}-Y_{i}\right|}{n}\\ \text{MSPE}=\frac{\sum_{i=1}^{n}{\left({\hat{Y}}_{i}-Y_{i}\right)}^{2}}{n} \end{array}$$

where n is validation data sample size, ${\hat{Y}}_{i}$is the predicted value, and *Y*_*i*_ is the observed value.

50% of the full dataset for each country were randomly selected as a validation dataset. The two statistics were used to measure how well the original models estimated on estimation data predict the validation data. The smaller the value of MAD and MSPE represents the more desirable model which fits the data as closely as possible[24].

The mean household WTP can be calculated by aggregating the area beneath the demand curve.

$$\text{WTP}\left(\text{vaccine}\right)=\overset{\infty }{\underset{0}{\int }}{\text{e}}^{\beta_{\text{i}}{\text{x}}_{\text{i}}+\beta_{\text{p}}{\text{P}}_{\text{i}}}\text{dP}=-\frac{{\text{e}}^{\beta_{\text{i}}{\text{x}}_{\text{i}}}}{\beta_{\text{p}}}$$

where β_p_ is the estimated coefficient for price and β_i_ is an array of the estimated coefficients for the other independent variables. The median WTP was also calculated by estimating the price at which an estimated 50% of the population would purchase vaccines. Parametric estimates of WTP are sensitive to the choice of distribution and functional forms of household demand. Non-parametric models, Turnbull lower bound and Kristrom’s midpoint models[13,27–29], were also estimated. The advantages of non-parametric models are in their simplicity and transparency. The Turnbull estimator does not impose any statistical assumptions about how WTP is distributed[13], and is considered to be a conservative measure. The Kristrom’s midpoint estimator assumes that the distribution between bid points is symmetrical[15]. Both models provide a useful comparison of mean and median WTP with the parametric WTP measures.

### Ethical considerations

The contingent valuation studies and survey questionnaires were approved by the ethical review committees in three countries (National Institute of Hygiene and Epidemiology in Vietnam, Faculty of Tropical Medicine, Mahidol University in Thailand, Universidad de Antioquia in Colombia), as well as Ministry of Health in three countries and Institutional Review Board of the International Vaccine Institute. Written informed consent was obtained prior to conducting interviews and respondents were informed that they could terminate interviews at any time. Respondents received compensation for their time.

## Results

Some respondents rejected the hypothetical scenario presented, and these respondents were dropped from our analysis. We identified such respondents based on their reasons for declining the vaccine, specifically those who reported that they believed that vaccines are not safe or that vaccines would cause some side effects. Only 1.5%, 2%, and 0.25% out of the 400 respondents rejected our hypothetical vaccine scenarios in Vietnam, Thailand, and Colombia, respectively.

To develop conservative WTP estimates, we also attempted to identify potential yea-sayers, i.e. people who reported unrealistically high WTP for vaccines. Potential yea-sayers were defined as those for whom the product of reported demand and offered vaccine price was greater than twice their reported monthly household income across all price points. All of the yea-sayers were identified at the highest price point. Because the highest price point in each country was designed to see the choke effect, it seemed unrealistic when respondents were willing to purchase vaccines for all members by paying a sum of more than twice their entire monthly household income. These responses (2%, 3.3%, and 1.8% in Vietnam, Thailand, and Colombia, respectively) were considered as outliers and dropped from our analysis.

### Household characteristics

Table 1 shows household characteristics for each study site. Average respondent age is from 37 to 47 years, and average household size is around 5 members. Most of the respondents are females. In order to make sure that their responses reflect the decisions made at the household, the respondents were asked who would be primarily involved in making decision for their household members. Over 80% of the respondents confirmed that they would be primarily involved in making vaccine purchasing decisions. The respondents had 6~9 years of median school education in Vietnam and Colombia, and 1~5 years of median school education in Thailand. The self-reported mean household income per month is $351, $788, and $367 in Vietnam, Thailand, and Colombia, respectively. For all three countries, more respondents thought that dengue fever is serious for children than respondents who thought so for adults, although the differences are not significant. Approximately 35%, 60%, and 87% of the respondents in Vietnam, Thailand, and Colombia said that their children would likely contract dengue in the next five years. In Vietnam and Thailand, about 28% of the respondents reported that at least one member of their household had contracted dengue fever in the past, compared to 10% in Colombia. Around 52%, 47%, and 27% of the respondents in Vietnam, Thailand, and Colombia mentioned that they know someone who had dengue fever in their neighborhoods. In response to past vaccine purchase history, 68% and 13% of the respondents in Vietnam and Colombia answered that they had previously purchased other vaccines. This question was not asked in Thailand. Over 99% of the total respondents from all three countries correctly answered questions designed to test their understanding of vaccine duration and efficacy.

**Table 1:** Descriptive statistics.

Parameter	Vietnam n = 386 (SD)	Thailand n = 379 (SD)	Colombia n = 392 (SD)
Average respondent age	39.26 (8.28)	47.36 (13.89)	37.49 (11.53)
Average household(HH) size	4.82 (1.6)	5.16 (1.74)	5.13 (2.03)
% of male respondents in the samples	22.02% (0.41)	20.31% (0.4)	7.91% (0.27)
% of respondents being a primary decision maker	84.97% (0.36)	84.7% (0.36)	90.61% (0.4)
Average number with Age < 1	0.1 (0.32)	0.04 (0.19)	0.08 (0.29)
Average number with 1 ≤ Age < 5	0.34 (0.56)	0.38 (0.57)	0.49 (0.63)
Average number with 5 ≤ Age < 15	0.91 (0.75)	1.05 (0.85)	1.29 (1.06)
Average number with 15 ≤ Age	3.46 (1.43)	3.69 (1.45)	3.27 (1.6)
Median education	6 to 9 years of school	1 to 5 years of school	6 to 9 years of school
% of respondents with no education	3% (0.17)	3.96% (0.2)	7.14% (0.26)
% of respondents with education 1–12 years, vocational	85.75% (0.35)	91.56% (0.28)	92.1% (0.27)
% of respondents with education higher than university	11.25% (0.32)	4.49% (0.21)	0.77% (0.09)
Monthly household income (USD)	350.74 (253.04)	788.49 (954.7)	366.84 (238.92)
Dengue is serious for children (%)	92.49% (0.26)	60.69% (0.49)	97.96% (0.14)
Dengue is serious for adults (%)	78.24% (0.41)	48.02% (0.5)	93.88% (0.24)
Likely to contract dengue for respondent (%)	32.12% (0.47)	44.06% (0.5)	85.71% (0.35)
Likely to contract dengue for children (%)	34.97% (0.48)	59.89% (0.49)	86.73% (0.34)
Previous dengue case in household (%)	28.24% (0.45) (3 deaths)	28.5% (0.45)	9.95% (0.3) (1 death)
Dengue in personal acquaintance (%)	51.81% (0.5)	46.97% (0.5)	26.53% (0.44)
Practice dengue vector control (%)	99% (0.1)	98.15% (0.13)	53.06% (0.5)
Prevent standing water (%)	26.68% (0.44)	91.03% (0.29)	51.02% (0.5)
Pass efficacy understanding test (%)	99%	99%	100%
Fraction who have purchased prior vaccine	67.88%(paid USD 0.12–70.84)	Not asked	13.01%(paid USD 1.27–265.15)

### Household demand analysis

Table 2 summarizes the raw data for average household vaccine demand as a function of price and efficacy. Vaccine demand decreases with price in all three countries. We did not find any significant difference in demand between the 70% and 95% efficacy scenarios.

**Table 2:** Household demand for vaccines by price and efficacy (average number of members for whom household would purchase vaccine based on price for three-dose series, raw data).

Vietnam			Thailand			Colombia		
Price	Household demand		Price	Household demand		Price	Household demand	
US$	70%	95%	US$	70%	95%	US$	70%	95%
2.93	2.70	2.90	1.97	3.79	4.13	1.62	4.25	3.97
11.70	2.39	2.49	9.84	2.80	3.68	32.47	1.40	1.78
21.94	1.77	2.23	29.52	2.77	3.00	81.16	0.85	1.38
43.88	1.80	1.64	59.03	2.58	2.25	162.33	0.53	0.56
268.17	0.56	0.50	491.94	0.52	0.39	486.98	0.36	0.21

**Fig 2:**
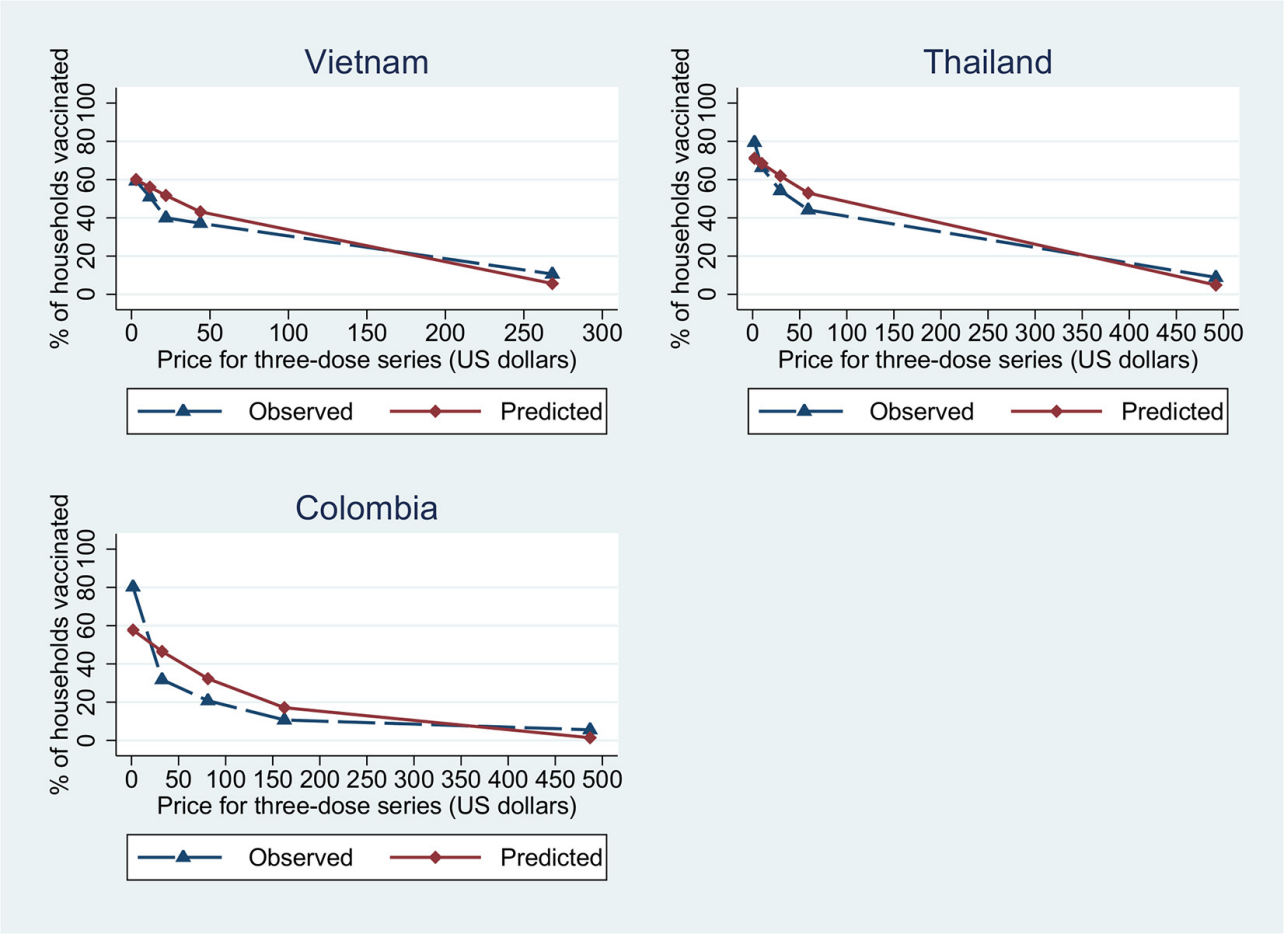
Fraction of household members who get vaccinated.

The regression results are shown in Table 3. Type 2 includes all possible covariates, while type 1 is a parsimonious model which includes only non-attitudinal variables. As expected, the price variable is statistically significant at the 1% level and has a negative sign. Income per capita (in log form) is also highly significant across countries with positive signs indicating that vaccine demand increases with income. The respondent age variable is inversely related to the likelihood that a respondent would purchase the vaccine in Vietnam, but this variable is not significant in Thailand and Colombia. The relationship between education and demand was not consistent across countries. Compared to no education, respondents with some education had significantly greater demand in Vietnam, but lower demand in Colombia. The coefficient was positive, but not significant in Thailand. The interaction between earning and price is positive and significant, meaning that respondents with a higher income can afford a more expensive vaccine. Neither the perceived seriousness of dengue nor the likelihood of contracting the disease in the next 5 years was a statistically significant determinant except the perceived seriousness in Colombia. Respondents in Vietnam who knew a person who had contracted dengue were more likely to be willing to purchase a vaccine. There is some evidence that respondents who had purchased other vaccines tend to demand more for our hypothetical dengue vaccine than those without previous vaccine purchase experience. The overall robustness of the model was examined by the MAD and MSPE statistics. In the field of transportation and accident analyses where the negative binomial models are more commonly used, 1.8 of MAD and 7.2 of MSPE were considered to be relatively small values for a mean dependent variable of 2.85[24,30]. Given that the mean value of the dependent variable in this study ranges from 1.54 to 2.66, the models for all three countries produced fairly satisfactory predictive performance. In particular, MSPE values are closer to 1 for both short and long models in Vietnam, meaning that the model fits the data better than the other countries. While the long model is preferred over the short model in Vietnam, the short models fit the data better for Thailand and Colombia. Fig 2 depicts the observed and predicted fractions of household members vaccinated by price.

**Table 3:** Factors affecting household demand for dengue vaccine (regression results).

Independent variables	Vietnam n = 386		Thailand n = 379		Colombia n = 392	
	Type 1	Type 2	Type 1	Type 2	Type 1	Type 2
Price (LCU)	-4.49E-07 (6.50E-08)***	-4.53E-07 (6.13E-08)***	-0.0002 (1.89E-05) ***	-0.0002 (1.89E-05) ***	-4.35E-06 (7.46E-07) ***	-4.40E-06 (7.87E-07) ***
ln(income per capita)	0.153 (0.072)**	0.141 (0.069)**	0.186 (0.056) ***	0.181 (0.056) ***	0.393 (0.126) ***	0.403 (0.125) ***
Respondent's age (yrs)	-0.007 (0.003)**	-0.009 (0.003)***	-0.003 (0.003)	-0.003 (0.003)	0.001 (0.004)	0.001 (0.004)
Male	0.09 (0.047) *	0.079 (0.06)	0.03 (0.08)	0.022 (0.084)	-0.155 (0.398)	-0.085 (0.389)
Education 1(some second / vocational)	0.315 (0.105)***	0.221 (0.077)***	0.148 (0.186)	0.172 (0.184)	-0.342 (0.097) ***	-0.303 (0.114) ***
Education 2(university / post graduate)	0.364 (0.19)*	0.246 (0.136)*	0.184 (0.211)	0.202 (0.208)	-1.75 (1.285)	-1.628 (1.313)
Age < 1(the number of hh members)	0.05 (0.098)	0.014 (0.076)	-0.006 (0.178)	-0.038 (0.175)	-0.298 (0.223)	-0.275 (0.22)
1 ≤ Age < 5(the number of hh members)	-0.089 (0.047)*	-0.089 (0.038)\**	0.146 (0.057)**	0.141 (0.059)**	0.199 (0.066) ***	0.203 (0.068) ***
5 ≤ Age < 15(the number of hh members)	-0.007 (0.055)	-0.019 (0.061)	0.033 (0.052)	0.036 (0.051)	0.043 (0.035)	0.043 (0.036)
15 ≤ Age(the number of hh members)	0.042 (0.046)	0.049 (0.041)	0.115 (0.028) ***	0.116 (0.029) ***	0.129 (0.037)***	0.121 (0.037) ***
Interaction (earning*price) (LCU)	1.47E-14 (2.28E-15) ***	1.51E-14 (2.26E-15) ***	9.24E-10 (1.09E-10) ***	9.05E-10 (1.12E-10) ***	8.84E-13 (2.78E-13)***	8.89E-13 (2.66E-13) ***
Efficacy	-	0.004 (0.002)*	-	0.001 (0.003)	-	0.004 (0.004)
Know previous dengue cases	-	0.07 (0.024) ***	-	-0.054 (0.061)	-	0.079 (0.084)
Dengue Seriousness	-	-0.00027 (0.041)	-	0.011 (0.043)	-	0.175 (0.093)*
Serious in the future	-	0.056 (0.038)	-	-0.007 (0.039)	-	0.102 (0.117)
Prevent standing water	-	0.096 (0.036) ***	-	0.229 (0.173)	-	-0.086 (0.059)
Previous vaccine purchase	-	0.194 (0.08)**	n/a	n/a	-	0.097 (0.114)
Constant	-1.377 (1.064)	-1.662 (0.962)*	-0.8 (0.576)	-1.066 (0.623)*	-3.921 (1.656)**	-5.041 (1.869) ***
MAD	1.09	1.07	1.51	1.53	1.23	1.25
MSPE	1.95	1.92	3.72	3.86	2.73	2.91

* Significance at the 10% level

** at the 5% level

*** at the 1% level.

Robust standard errors in parentheses

Table 4 shows parametric and non-parametric mean WTP estimates for the three-dose series described in the hypothetical scenario. The mean WTP per dose is included in parentheses. We did not generate separate estimates by vaccine efficacy/duration since the difference in vaccine demand was not statistically significant. The conservative Turnbull-lower bound mean WTP is $42.3 ($14.1 per dose) for a 3 dose-series vaccine in Vietnam, $68.8 ($22.9 per dose) in Thailand, and $48 ($16 per dose) in Colombia. The parametric mean WTP estimates lie in between Turnbull lower bound and Kristrom midpoint values except Colombia. The mean WTP in Thailand is higher than for the other two countries.

**Table 4:** Mean willingness-to-pay per person.

Countries	Non-parametric		Parametric	
	Mean WTP, TB^a^ (/dose)	Mean WTP, KR^b^ (/dose)	Mean WTP, type 1 (/dose)	Mean WTP, type 2 (/dose)
Vietnam (n = 386)	$42.26 ($14.09)^c^	$75.15 ($25.05)	$73.39 ($24.46)	$73.4 ($24.47)
Thailand (n = 379)	$68.79 ($22.93)	$155.82 ($51.94)	$141.77 ($47.26)	$141.76 ($47.25)
Colombia (n = 392)	$47.99 ($16)	$72.24 ($24.08)	$91.34 ($30.45)	$90.84 ($30.28)

^a^ Turnbull lower bound estimate

^b^ Kristrom midpoint estimate

^c^ Values in parentheses show price per dose

Table 5 summarizes the median WTP estimates for both parametric and non-parametric models. The median WTP is calculated based on the price in which an estimated 50% of the population would purchase vaccines. Median estimates tend to be less sensitive to the unexpected responses and functional form assumptions than mean estimates, as long as the empirical 50^th^ percentile value lies in between the lowest and the highest price points[13]. For all three countries, the observed median WTP estimates fall between the lowest price and the highest price offered in the surveys. In the case of non-parametric models, the point estimates were linearly interpolated. The parametric median WTP is $26.4 ($8.8 per dose) for a 3 dose-series vaccine in Vietnam, $70 ($23.4 per dose) in Thailand, and $23 ($7.7 per dose) in Colombia. The median WTP in Thailand is again higher than the other countries.

**Table 5:** Median willingness-to-pay per person.

Countries	Non-parametric		Parametric	
	Median WTP, TB^a^ (/dose)	Median WTP, KR^b^ (/dose)	Median WTP, Type 1 (/dose)	Median WTP, Type 2 (/dose)
Vietnam (n = 386)	$12.59 ($4.20)^c^	$18.21 ($6.07)	$26.38 ($8.79)	$26.13 ($8.71)
Thailand (n = 379)	$41.86 ($13.95)	$140.95 ($46.98)	$70.31 ($23.43)	$69.78 ($23.26)
Colombia (n = 392)	$20.86 ($6.95)	$41.85 ($13.95)	$23 ($7.67)	$22.6 ($7.53)

^a^ Turnbull lower bound estimate

^b^ Kristrom midpoint estimate

^c^ Values in parentheses show price per dose

**Fig 3:**
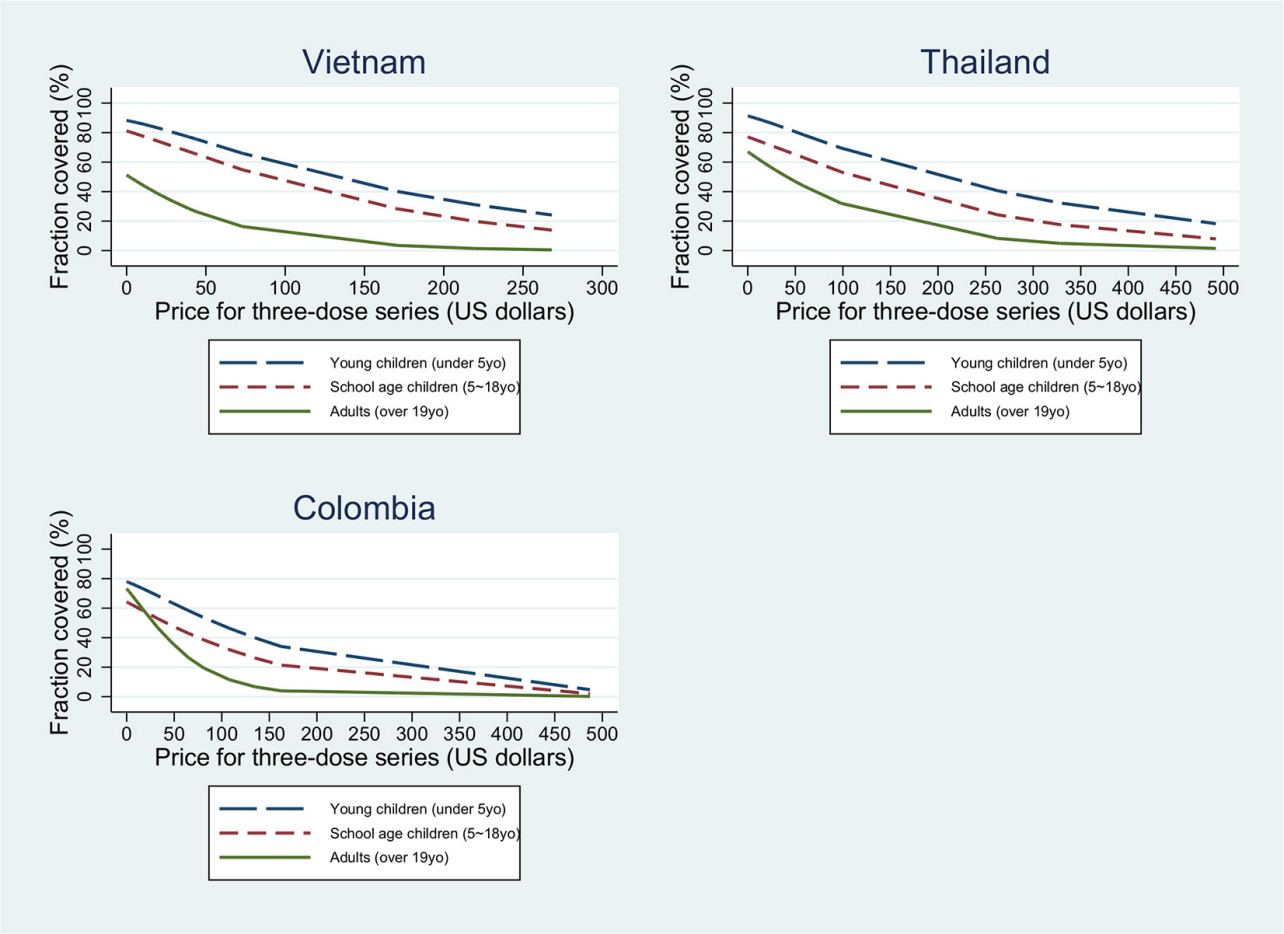
Predicted coverage rates for different age groups.

It is also possible to create separate sub-models and estimate vaccine demand for different age groups. We divided households into three groups: young children (age under 5 years), school age children (age 5–18 years), and adults (age over 19 years). Fig 3 shows the predicted coverage as a function of price for the three age groups. For all three countries, the predicted fractions of young children vaccinated are higher than those for the other age groups at any price, suggesting that respondents place more value on vaccinating young children than school age children and adults.

## Discussion

This study provides insight into the private economic benefits of potential dengue vaccine in three countries. The median WTP per household member was $26.1 ($8.7 per dose) in Nha Trang, Vietnam, $69.8 ($23.3 per dose) in Ratchaburi, Thailand, and $22.6 ($7.5 per dose) in Medellin, Colombia. Our models showed that household demand for the dengue vaccine is sensitive to price and income, suggesting that respondents took the hypothetical purchasing scenario seriously. These results suggest the possibility that a private market for dengue vaccines exists in these three countries and that sales may be robust if vaccine prices are lower than the median estimates from our study. Since respondents were not bound by their stated purchasing decisions, it is possible they may not act as they reported.

There was a relatively large fraction of respondents who were willing to purchase vaccines at high price points in Thailand (11%) compared to those in Vietnam (9%) and Colombia (3%). One explanation for higher WTP in Thailand is that the mean reported household income in Thailand is almost two times greater than that in Vietnam and Colombia; therefore, Thai respondents had more purchasing power. In addition, we were not able to employ the *time-to-think* research design in Thailand due to budget and logistical constraints. Other researchers have found that people tend to report lower WTP when they have more time to think about a new vaccine product and their budget constraints[14–17]. It is worth noting that this study did not attempt to test the validity of the *time-to-think* approach. Rather, the study was designed based upon evidence from the *time-to-think* option used in the previous studies [17]. The *time-to-think* approach allowed respondents to think carefully about their budget constraints and may more accurately reflect their willingness-to-pay for the vaccine. While the absence of the *time-to-think* option in Thailand may contribute to higher WTP compared to Vietnam and Colombia, the exact magnitude could not be measured in this study. The detailed methodology and comparison for the *time-to-think* approach were extensively discussed by Cook J et al.[17].

It should be noted that two parameter estimates among the significant determinants differ in sign by country: education 1 and age group 2. While it is common for regression coefficients to have the same direction towards the underlying concept in the similar circumstance, some of the socio-economic variables may behave differently across countries to explain variance of the dependent variable (vaccine demand) due to the diverse contexts of local specific situations. Ideally, our study samples would be more heterogeneous and more representative of the entire countries; however, we were limited to performing the studies in locations where epidemiologic studies were being conducted at the same time. As a result, these results may not be generalizable beyond the communities in which the studies were conducted. In comparison with previous studies, a study from Philippines suggests a median WTP of $60 for a 10 year efficacy scenario of dengue vaccine[31], and a study from Indonesia shows a median WTP of $1.94 for a fully efficacious dengue vaccine[32]. The estimates may differ depending upon income levels of study populations and previous experience in receiving other vaccines in the study communities (i.e. availability of free vaccines from local health centers, etc.).

The rise in dengue fever cases and the absence of dengue vaccines will likely cause governments to consider various types of effective means for controlling the disease. The contingent valuation study proposed here provides important information—how much people are willing to pay for a dengue fever vaccine to avoid the risk of getting infected. The WTP estimates provide quantification of the private benefit of disease reduction. Results can be incorporated into cost benefit analyses, which can inform prioritization of different health interventions at the national level. The study can also assist decision makers to understand how much of population can be covered by subsidizing dengue vaccines when implementing the nationwide campaigns and can help inform how households would allocate vaccines across age groups given household budget constraints. Further, the WTP study provides vaccine manufacturers a better picture of people’s perceptions of dengue fever and dengue vaccines.

## Supporting Information

S1 Text Overdispersion. (DOCX)

S2 Text Description for dengue fever CV scenario. (DOCX)

S1 Fig Distributions of observed, Poisson, and negative binomial. (TIF)

S2 Fig Diagram for vaccine effectiveness. (TIF)
