# Correction: A Multi-country Study of the Household Willingness-to-Pay for Dengue Vaccines: Household Surveys in Vietnam, Thailand, and Colombia

**DOI:** 10.1371/journal.pntd.0003886

**Published:** 2015-06-24

**Authors:** Jung-Seok Lee, Vittal Mogasale, Jacqueline K. Lim, Mabel Carabali, Chukiat Sirivichayakul, Dang Duc Anh, Kang-Sung Lee, Vu Dinh Thiem, Kriengsak Limkittikul, Le Huu Tho, Ivan D. Velez, Jorge E. Osorio, Pornthep Chanthavanich, Luiz J. da Silva, Brian A. Maskery

There is an omission in the Acknowledgements section. The correct Acknowledgments statement is as follows: We would like to thank all members in our local sites for their support and collaboration. We are highly grateful to the Dengue Vaccine Initiative (DVI) team and would like to acknowledge the excellent team work. The International Vaccine Institute (IVI) also acknowledges the Governments of the Republic of Korea and Sweden for their core support to IVI.
