# Correction: A Multi-country Study of the Household Willingness-to-Pay for Dengue Vaccines: Household Surveys in Vietnam, Thailand, and Colombia

**DOI:** 10.1371/journal.pntd.0004070

**Published:** 2015-09-17

**Authors:** Jung-Seok Lee, Vittal Mogasale, Jacqueline K. Lim, Mabel Carabali, Chukiat Sirivichayakul, Dang Duc Anh, Kang-Sung Lee, Vu Dinh Thiem, Kriengsak Limkittikul, Le Huu Tho, Ivan D. Velez, Jorge E. Osorio, Pornthep Chanthavanich, Luiz J. da Silva, Brian A. Maskery

There are errors in the Competing Interests section and in the affiliations listed for 12^th^ author, Jorge E. Osorio.

The complete, correct Competing Interests information is as follows: Author Jorge E. Osorio is fully employed by Takeda Vaccines. He was a co-founder of lnviragen, Inc. a company that licensed the live attenuated dengue vaccine candidate from the CDC and that was later acquired by Takeda. He has actively worked in the preclinical and clinical development of this dengue vaccine and is also a patent holder on chemical formulations that provide increased stability to the vaccine. He has a direct financial interest in seeing the dengue vaccine developed, approved and commercialized.

The correct affiliations for Jorge E. Osorio are as follows: Department of Pathobiological Sciences, University of Wisconsin, Madison, Madison, Wisconsin, United States of America, and Takeda Vaccines, Inc.
