# Supplementary material for: A Multi-country Study of the Household Willingness-to-Pay for Dengue Vaccines: Household Surveys in Vietnam, Thailand, and Colombia
Source: PLoS Negl Trop Dis. 2015 Jun 1;9(6):e0003810. doi: 10.1371/journal.pntd.0003810 (PMC4452082; doi:10.1371/journal.pntd.0003810)
Supplement: S1 Text — (DOCX) [file pntd.0003810.s001.docx]

**S1. Overdispersion**

Prior to analyzing the parametric models, the Z-score test and the boundary likelihood ratio test were carried out. The two tests can be written as[[22](#_ENREF_22)]

$$Z_{i}=\frac{\left( n_{i}-\mu_{i} \right)^{2}-n_{i}}{\mu_{i}\sqrt{2}}$$

$$LR=-2\left( \mathcal{L}_{P}-\mathcal{L}_{\mathrm{NB}} \right)$$

where $\mu_{i}$ is predicted values, $\mathcal{L}_{P}$ is a Poisson log-likelihood estimate, and $\mathcal{L}_{\mathrm{NB}}$ is an negative binomial log-likelihood estimate. The null hypotheses of the two tests are that there is no overdispersion in a dataset. The null hypotheses were rejected for Thailand and Colombia but not for Vietnam. S1 Fig. compares the probability of vaccine demand among observed values, Poisson distribution, and negative binomial distribution. It is difficult to say that there is any significant difference between Poisson and negative binomial in Vietnam, but it is clear to see that the negative binomial distribution performs much better than the Poisson distribution in comparison with the observed probability in Thailand and Colombia. This visualization is also consistent with the results from the two test statistics. Thus, the negative binomial model was adopted for Thailand and Colombia, and the Poisson model was employed for Vietnam.
