# Supplementary material for: A Multi-country Study of the Household Willingness-to-Pay for Dengue Vaccines: Household Surveys in Vietnam, Thailand, and Colombia
Source: PLoS Negl Trop Dis. 2015 Jun 1;9(6):e0003810. doi: 10.1371/journal.pntd.0003810 (PMC4452082; doi:10.1371/journal.pntd.0003810)
Supplement: S2 Text — (DOCX) [file pntd.0003810.s002.docx]

**S2. Description for dengue fever CV scenario**

Interviewers read out the following contents and showed the pictures to the respondents:

“For many years, doctors and scientists have been working to invent a new vaccine that protects against dengue fever. Imagine that they have successfully developed a new vaccine that can prevent people from getting dengue fever. We'd like to know what you would do if the new dengue fever vaccine was available for sale at a convenient location like a vaccination camp or vaccination clinic. This new vaccine could be given to individuals to prevent them from having dengue fever in the future. It could not be used to treat someone who currently has dengue fever. This vaccine would require three doses. The vaccine would be available for all ages including newborn babies. However, pregnant women would not be eligible to receive the vaccine.

Suppose that this vaccine has no side effects, and is safe, that is, after you were vaccinated you would have no chance to get dengue fever from the vaccine. Suppose that the vaccine requires an injection, which may be painful for a few days. Assume that three doses of the vaccine would be required, and that each dose should be administered six months after the previous dose. Suppose that taking the all three doses of dengue fever vaccine would be required to achieve [70%/95% effectiveness for 10/30 years].

**Vaccine effectiveness**

Now I want to explain exactly what I mean when I say the vaccine would be [70%/95%] effective.

Suppose that each of these little blue or red figures (Enumerator: show S2 Fig.) represents a person (Enumerator: point out the circle). The 100 figures inside this circle represent 100 persons who have taken the vaccine, while the figures outside the circle represents those who have not taken the vaccine. The dengue fever vaccine is not 100% effective; that is the vaccine is only (70%/95%) effective. Therefore, of the 100 people taking the vaccine in the circle, there will be (70/95) of the people who have taken the vaccine that are protected (i.e., the vaccine works for them) for a period of 10/30 years. The blue figures inside this circle represent these people.

The rest of the people (the red ones inside the circle) who have been vaccinated [30/5] will not be protected against dengue fever even though they have taken the vaccine, because the vaccines did not work for them. They will still be at risk of getting dengue fever just like they were before they got the vaccine or just like the people outside the circle who haven't received vaccines.

The people who receive dengue fever vaccine will not be able to know if the vaccine works for them. Of course, we don't know who would actually get dengue fever. A red person outside the circle who has not taken a vaccine may not be infected.

Now I am going to ask you some questions to make sure that the information I told you is clear.”
