# Supplementary figures and images for: A Multi-country Study of the Household Willingness-to-Pay for Dengue Vaccines: Household Surveys in Vietnam, Thailand, and Colombia
Source: PLoS Negl Trop Dis. 2015 Jun 1;9(6):e0003810. doi: 10.1371/journal.pntd.0003810 (PMC4452082; doi:10.1371/journal.pntd.0003810)

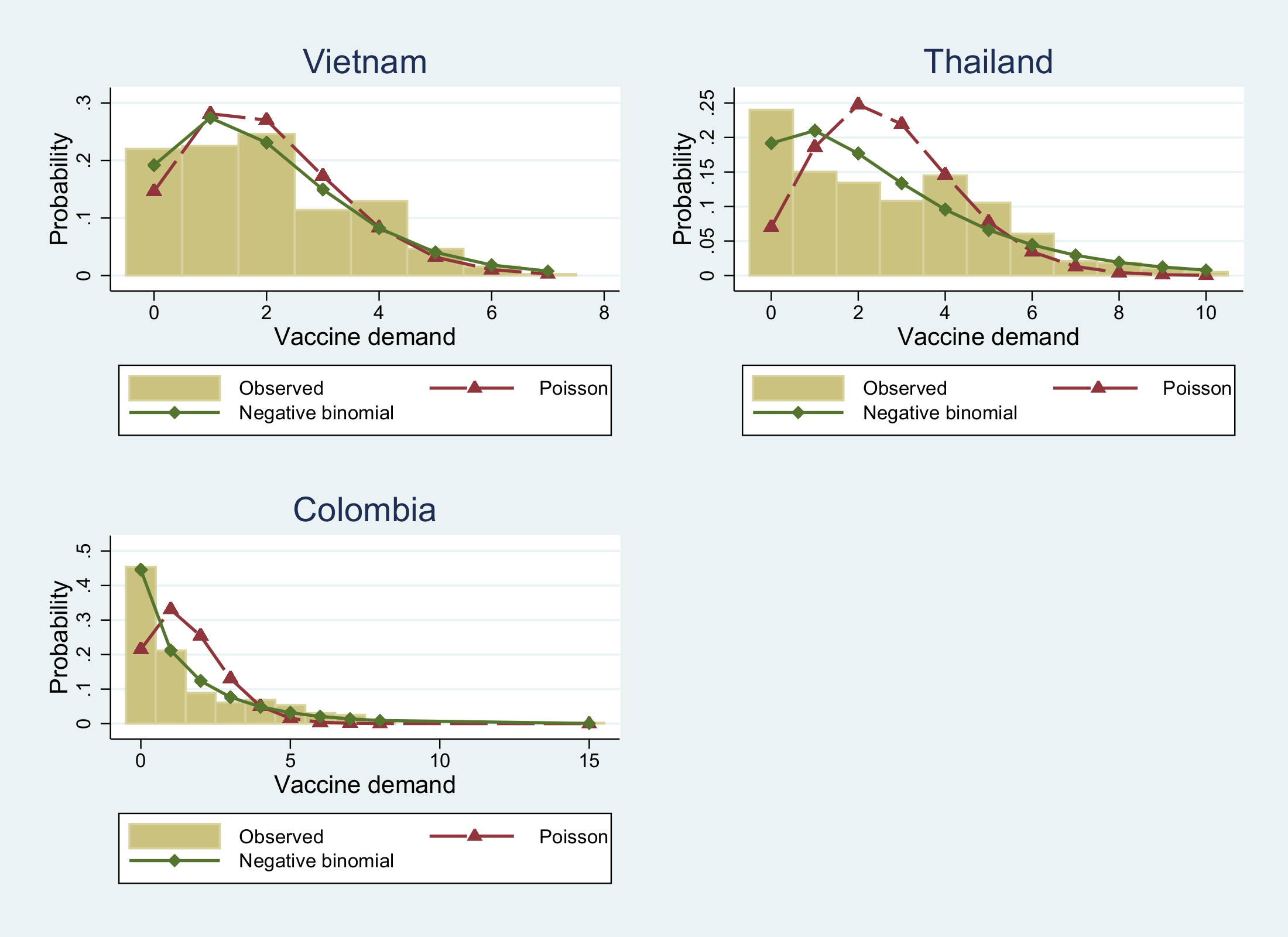

Supplement: S1 Fig — (TIF) [file pntd.0003810.s003.tif]

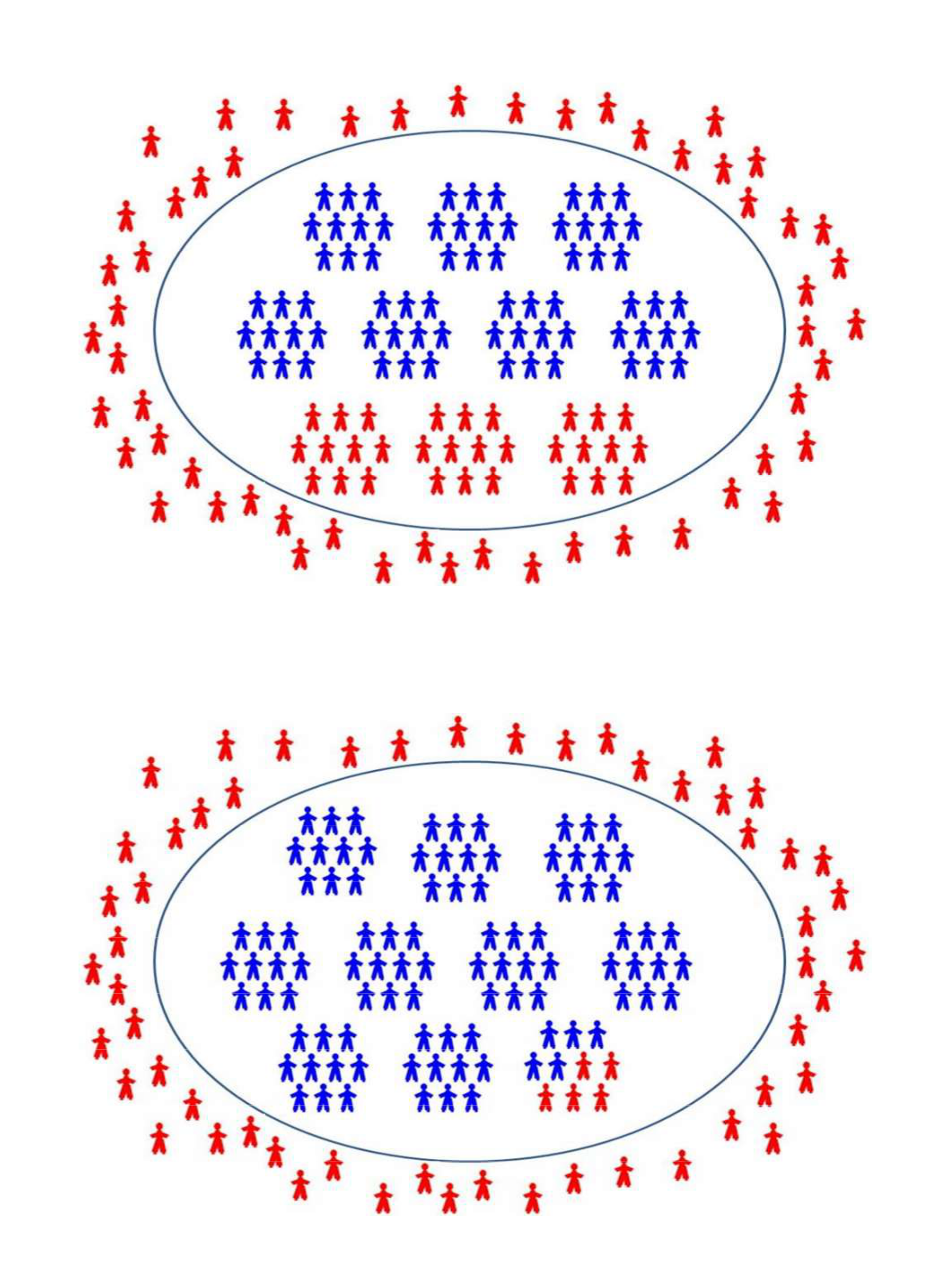

Supplement: S2 Fig — (TIF) [file pntd.0003810.s004.tif]
